# Formulation of Gamma-Oryzanol Encapsulated Nanoparticles and Their Modulation Effects on Inducible Nitric Oxide Synthase and Nitric Oxide in LPS-Stimulated RAW 264.7 Macrophages

**DOI:** 10.3390/pharmaceutics18030365

**Published:** 2026-03-14

**Authors:** Kornvipa Settakorn, Chuda Chittasupho, Weerasak Samee, Nut Koonrungsesomboon, Mingkwan Na Takuathung

**Affiliations:** 1 Department of Pharmacology, Faculty of Medicine, Chiang Mai University, Chiang Mai 50200, Thailand; kornvipa.s@cmu.ac.th (K.S.); nut.koonrung@cmu.ac.th (N.K.); 2 Clinical Research Center for Food and Herbal Product Trials and Development (CR-FAH), Faculty of Medicine, Chiang Mai University, Chiang Mai 50200, Thailand; 3 Department of Pharmaceutical Sciences, Faculty of Pharmacy, Chiang Mai University, Chiang Mai 50200, Thailand; chuda.c@cmu.ac.th; 4 Department of Pharmaceutical Chemistry, Faculty of Pharmacy, Srinakharinwirot University, Nakhon Nayok 26120, Thailand; weerasak@g.swu.ac.th

**Keywords:** gamma-oryzanol, nanoparticles, nitric oxide, inducible nitric oxide synthase, inflammation, RAW 264.7 macrophage cells

## Abstract

**Background/Objectives**: Gamma-oryzanol (ORZ), a bioactive compound extracted from rice bran oil, has health-promoting properties but limited therapeutic use due to poor stability and bioavailability. This study aimed to synthesize gamma-oryzanol-encapsulated nanoparticles (ORZ-NPs) and investigate their anti-inflammatory effects in lipopolysaccharide-stimulated RAW 264.7 macrophages. **Methods**: ORZ-NPs were synthesized via nanoprecipitation and characterized by dynamic light scattering and transmission electron microscopy. ORZ content was assessed using high performance liquid chromatography. In vitro release was determined using a dialysis method. Inducible nitric oxide synthase (iNOS) was assessed by Western blotting, nitric oxide (NO) by Griess assay, and tumor necrosis factor-alpha (TNF-α) and interleukin-6 (IL-6) by enzyme-linked immunosorbent assay. **Results**: ORZ-NPs exhibited spherical morphology with a mean particle size of 93.320 ± 2.027 nm, polydispersity index 0.149 ± 0.025, and zeta potential −22.400 ± 0.252 mV. ORZ remained stable for 90 days. In vitro release reached 70% at 24 h in PBS (pH 7.4). At 50 μg mL^−1^, ORZ-NPs significantly decreased iNOS and NO production (approximately 65% of control, *p* < 0.01), without affecting TNF-α or IL-6. **Conclusions**: ORZ-NPs demonstrate selective anti-inflammatory activities by suppressing iNOS and NO production while pro-inflammatory cytokines remain unaffected. These findings suggest a partial modulatory effect on the inflammatory signaling pathway.

## 1. Introduction

Traditionally, rice bran oil, derived from the outer layer of rice kernels, has been valued for its pharmaceutical and therapeutic benefits, particularly in Asian cultures, where it has been used for centuries in cooking and medicinal practices. It is a rich source of bioactive compounds, including gamma-oryzanol (ORZ), tocopherols, and phytosterols, all recognized for their significant health benefits [1]. Among these, ORZ stands out for its health-promoting properties, with recent studies highlighting its notable anti-inflammatory and antioxidant effects [2,3,4,5]. Its anti-inflammatory mechanisms involve the inhibition of key signaling pathways, including nuclear factor-kappa B (NF-κB) and mitogen-activated protein kinase (MAPK), which are pivotal regulators of inflammation [6,7,8]. Additionally, ORZ suppresses the overproduction of pro-inflammatory mediators and cytokines, thereby mitigating inflammatory responses and alleviating related conditions [9,10,11,12]. These properties make ORZ a promising candidate for combating chronic inflammation.

Despite its potential, the clinical application of ORZ is hindered by poor stability and low bioavailability, as it is highly susceptible to degradation under environmental conditions such as heat, light, and oxygen [13]. To address these limitations, encapsulating ORZ within nanoparticles (NPs) has emerged as a promising strategy [14]. NPs-based formulations not only improve the stability and bioavailability of ORZ but also enable controlled release, ensuring prolonged therapeutic effects [15]. By protecting ORZ from environmental degradation and enhancing its targeted delivery, NPs could potentially amplify its anti-inflammatory and antioxidant properties [16], making them a promising approach for managing chronic inflammation and related diseases.

Chronic inflammation, which underpins many diseases, provides a key therapeutic target for ORZ’s enhanced properties. Inflammation, a complex defense mechanism, plays a central role in the immune response to tissue injury and pathogen invasion [17]. This process involves the activation of intracellular inflammatory mediators, including signal transducer and activator for transcription-3 and NF-κB. These mediators enhance the production of pro-inflammatory cytokines such as tumor necrosis factor-alpha (TNF-α), interleukin-6 (IL-6), and interleukin-1 (IL-1) [18]. While these cytokines are essential for acute inflammatory responses, their persistent overproduction can lead to chronic inflammation, a key contributor to various diseases such as cardiovascular diseases, atherosclerosis, diabetes, and cancer [19]. In addition, nitric oxide (NO) also plays a significant role in the inflammatory response. At sites of inflammation, large quantities of NO are produced by inducible nitric oxide synthase (iNOS) in activated tissue cells and infiltrating leukocytes [20]. NO serves as a signaling molecule with dual roles. Under normal physiological conditions, it shows anti-inflammatory effects, whereas excessive NO production under pathological conditions contributes to inflammation and tissue damage [21]. Consequently, regulating inflammatory mediators such as cytokines and NO represent a promising strategy for alleviating inflammation and preventing its progression to chronic diseases. Encapsulation of ORZ into NPs offers a potential solution for achieving this regulation, combining enhanced stability with targeted therapeutic action against inflammatory mediators.

This study aimed to investigate the anti-inflammatory properties of ORZ encapsulated in NPs (ORZ-NPs) in lipopolysaccharide (LPS)-stimulated RAW 264.7 macrophages, a widely used model for investigating inflammation. The effects of the ORZ-NPs (0–50 μg mL^−1^) were analyzed on key inflammatory mediators, including iNOS and NO, as well as key pro-inflammatory cytokines, including TNF-α and IL-6. Furthermore, the long-term stability of the ORZ-NPs was assessed through physical characterization and chromatographic analysis. These findings highlight the potential of ORZ-NPs to partially modulate the inflammatory signaling pathway.

## 2. Materials and Methods

### 2.1. Chemicals and Reagents

ORZ was obtained from Toronto Research Chemicals (Toronto, ON, Canada). Phosphatidylcholine and Poloxamer 407 were sourced from Chanjao Longevity Co., Ltd. (Bangkok, Thailand), while polyethylene glycol (PEG) 400 was acquired from Dow Inc. (Midland, MI, USA). RAW 264.7 macrophage-like cells were obtained from CLS Cell Lines Service (Eppelheim, Germany). Dulbecco’s Modified Eagle Medium (DMEM), L-glutamine, fetal bovine serum, penicillin/streptomycin, LPS, mouse anti-iNOS primary antibody, and mouse anti-β-actin primary antibody were purchased from Sigma-Aldrich (St. Louis, MO, USA). The Clarity^TM^ Western enhanced chemiluminescence (ECL) substrate, protein assay dye, and Tris-buffered saline with Tween 20 (TBST) were purchased from Bio-Rad Laboratories (Hercules, CA, USA). Mammalian cell lysis buffer was purchased from GoldBio (St. Louis, MO, USA). Nitrocellulose membranes and Pierce^TM^ G2 Fast Blotter were purchased from Thermo Fisher Scientific (Waltham, MA, USA).

### 2.2. Formulation of ORZ-NPs

The ORZ-NPs were synthesized using the nanoprecipitation method [22]. Initially, 40 mg of ORZ was dissolved in 2 mL of 95% ethanol, followed by the addition of 30 mg of phosphatidylcholine, which was also dissolved in 2 mL of ethanol. The resulting 4 mL mixture was introduced dropwise into 20 mL of an aqueous phase containing 0.1% (*w*/*v*) Poloxamer 407 and 10% (*v*/*v*) PEG 400 at a flow rate of 8 mL per hour, under constant stirring at 550 rpm. The mixture was stirred for an additional 2 h to ensure complete removal of residual ethanol. The synthesized ORZ-NPs were subsequently stored at 4 °C, 30 °C, and 45 °C for further characterization.

### 2.3. Physical Characterization of the ORZ-NPs

The particle size, polydispersity (PDI), and zeta potential of the ORZ-NPs were analyzed using dynamic light scattering (DLS) at a 173° scattering angle and 25 °C with a Zetasizer NanoSeries (Malvern Instruments, Malvern, UK). To assess the long-term stability of the ORZ-NPs, samples were stored at 4 °C, 30 °C, and 45 °C, protected from light, for 12 weeks, in accordance with the International Council for Harmonisation (ICH) guidelines of Technical Requirements for Pharmaceuticals for Human Use [23]. Measurements of particle size, PDI, and zeta potential were performed at designated time points (0, 1, 2, 3, 4, 8, and 12 weeks) to assess the impact of storage conditions on the physical properties of the ORZ-NPs.

### 2.4. Morphological Characterization of ORZ-NPs

The morphology of the ORZ-NPs was analyzed using transmission electron microscopy (TEM) (Hitachi HT7700, Hitachinaka, Ibaraki, Japan). A Formvar-coated copper grid (300 mesh, Sigma-Aldrich, USA) was immersed in the nanoparticle suspension for 1 min to facilitate particle adsorption onto the grid surface. The excess solution was carefully removed, and the grid was subsequently stained with 2% (*w*/*v*) uranyl acetate for 30 s to enhance contrast. The grid was then air-dried in a desiccator to eliminate moisture. TEM imaging was conducted at 75 kV following the drying process, and NPs were captured at a magnification of 50,000×.

### 2.5. Quantification and Chemical Stability of ORZ-NPs

The NPs suspension, containing both encapsulated and free ORZ, was first diluted 10-fold with ethanol. The mixture was then sonicated for 20 min to ensure uniform dispersion and prevent aggregation. Following this, the suspension was centrifuged at 8000 rpm for 10 min. The resulting pellet, presumed to contain nanoparticles, was collected for further analysis. The supernatant, which contained the unencapsulated ORZ, was subsequently filtered through a 0.45 μm membrane filter to eliminate any residual particulates. To recover any nanoparticles that may have been retained on the filter, the membrane was washed and resuspended in the solvent.

ORZ-NPs were diluted 10-fold with 95% ethanol and filtered through a 0.45 µm nylon membrane prior to analysis using reverse-phase high-performance liquid chromatography (HPLC). The HPLC system (Agilent, Santa Clara, CA, USA), equipped with the 1260 Infinity II quaternary pump, autosampler, multi-column thermostat, and photodiode array detector, was used to quantify ORZ in NPs. Chromatographic separation was conducted using an ACE ^®^ C18-AR column (4.6 mm × 250 mm, 5 µm particle size) paired with a Phenomenex C18 guard column (4 mm × 3 mm, 5 μm particle size). The mobile phase, consisting of acetonitrile and methanol (95:5, *v*/*v*), was degassed before use and freshly prepared for each analysis. Samples volumes of 10 µL were eluted isocratically at a flow rate of 2.0 mL per minute, with the column temperature maintained at 35 °C. The ORZ yield was monitored at 320 nm. The average retention times for the ORZ components–cycloartenyl ferulate, methylenecycloartanyl ferulate, campesteryl ferulate, and β-sitosteryl ferulate–were around 16.3, 18.2, 20.9, and 23.4 min, respectively. The method was validated in ORZ-NPs solution according to the ICH guidelines, assessing linearity, selectivity, accuracy, precision, limit of detection, and limit of quantification.

The stability of the ORZ-NPs was assessed by determining the percentage of ORZ retained over time using HPLC analysis. Samples were evaluated at four time points: day 0, 30, 60, and 90, under three storage conditions—at 4 °C, 30 °C, and 45 °C. The percentage of ORZ retention was calculated using the following formula: [(concentration of ORZ in NPs/concentration of ORZ in freshly prepared NPs at day 0) × 100%].

### 2.6. In Vitro Release of ORZ from NPs

In vitro release of ORZ from NPs and ORZ powder was evaluated using a modified Transwell^®^ insert method at 37 °C, based on a previously reported protocol [22]. Twelve-well Transwell^®^ plates with polycarbonate membrane inserts (0.4 μm pore size) (Corning Incorporated, Kennebunk, ME, USA) were used for this study. ORZ-NPs (200 μL, 2 mg mL^−1^) under various conditions, including phosphate-buffered saline (PBS) pH 7.4, culture medium without additional supplements, and acetate buffer pH 5.5, were placed on the semi-permeable membrane in the upper chamber of the Transwell^®^. The receptor compartment was filled with 1.0 mL of preheated PBS (pH 7.4), culture medium without additional supplements, or acetate buffer (pH 5.5). Whereas ORZ powder (200 μL, 2 mg mL^−1^) were assessed only in PBS pH 7.4. At predetermined intervals (0.25, 0.5, 1, 2, 4, 6, 8, 12, and 24 h), 50 μL of the solution was withdrawn from the receptor compartment and replaced with an equal volume of fresh preheated PBS, culture medium without additional supplements, or acetate buffer to maintain sink conditions. To ensure complete dissolution of ORZ, 50 μL of dimethyl sulfoxide (DMSO) was added to each sample. The ORZ content was quantified using ultraviolet (UV)–visible spectrophotometry at a maximum wavelength of 333 nm, and the concentrations were calculated based on an ORZ standard curve.

### 2.7. Cell Culture

RAW 264.7 macrophage cells were cultured in DMEM supplemented with L-glutamine, 10% (*v*/*v*) heat-inactivated fetal bovine serum, and 1% (*v*/*v*) penicillin/streptomycin. The cells were grown in Corning^®^ 60 mm ultra-low attachment dishes and maintained at 37 °C in a humidified atmosphere containing 5% CO_2_ (Thermo Scientific, Waltham, MA, USA). Sub-culturing was performed every three days.

### 2.8. Cytotoxicity Testing

The cytotoxic effects of the ORZ-NPs or blank NPs against RAW 264.7 macrophages were assessed using a 3-(4,5-dimethylthiazol-2-yl)-2,5-diphenyltetrazolium bromide (MTT) assay [24]. Briefly, RAW 264.7 macrophage cells (2.5 × 10^3^ cells per well) were seeded and incubated for 24 h. Subsequently, the cells were exposed to various concentrations of ORZ-NPs or blank NPs, ranging from 0 to 200 μg mL^−1^ for 48 h. Then, the cells were incubated with MTT solution (0.5 mg mL^−1^) for 4 h. The formazan crystals were dissolved in 100 μL of DMSO. Absorbance at 560 nm was measured using a UV-visible spectrophotometer (BioTek’s Synergy™ H4, BioTek Instruments, Winooski, VT, USA). Cell viability was calculated relative to the LPS-induced control and expressed as a percentage of the control.

### 2.9. Quantification of iNOS Using Western Blotting Analysis

To investigate the effects of ORZ-NPs on LPS-induced inflammation, RAW 264.7 macrophage cells were pre-treated with the ORZ-NPs or blank NPs at concentrations of 25 and 50 µg mL^−1^ for 1 h and then exposed to LPS (1 µg mL^−1^). Afterwards, the cells were harvested and lysed, and protein concentration was determined using protein assay dye.

Cell lysates were separated by 10% (*w*/*v*) sodium dodecyl sulphate-polyacrylamide gel electrophoresis and subsequently transferred to nitrocellulose membranes using Pierce^TM^ G2 Fast Blotter. The membranes were incubated overnight at 4 °C with primary antibodies: mouse anti-iNOS (1:2000) or mouse anti-β-actin (1:10,000). After incubation, the membranes were washed twice with 0.3% (*v*/*v*) TBST and incubated for 2 h at room temperature with secondary antibodies: either goat anti-mouse IgG conjugated to peroxidase (1:10,000) or goat anti-rabbit IgG conjugated to peroxidase (1:10,000) in 0.1% (*v*/*v*) TBST. This was followed by five washes with 0.3% (*v*/*v*) TBST to remove unbound antibodies. Protein bands were visualized using an ECL detection method. The resulting bands corresponded to iNOS (131 kDa) and β-actin (42 kDa).

### 2.10. Determination of NO Production

RAW 264.7 macrophage cells (2.5 × 10^4^ cells per well) were seeded and cultured for 24 h. The cells were then treated with ORZ-NPs or blank NPs at 25 and 50 µg mL^−1^ concentrations for 1 h. Following treatment, 1 µg mL^−1^ of LPS was added to induce inflammation, and the cells were further incubated for an additional 24 h. The supernatant was collected to assess NO production using the Griess reagent assay, with absorbance measured at 540 nm [25].

### 2.11. Assessment of TNF-α and IL-6

The concentrations of TNF-α and IL-6 secreted into the culture medium were measured using a sandwich enzyme-linked immunosorbent assay (ELISA) kit (BioLegend ELISA MAX^™^ Deluxe Set, San Diego, CA, USA), following the manufacturer’s instructions. Briefly, RAW 264.7 macrophage cells were pre-treated with ORZ-NPs or blank NPs at 25 and 50 µg mL^−1^ concentrations for 1 h. Inflammation was then induced by adding LPS (1 µg mL^−1^), and the cells were further incubated for 24 h. After incubation, the culture medium was collected and analyzed using the ELISA assay. Absorbance was measured at 450 and 620 nm. The cytokine levels in the treated groups were compared to those in the LPS-induced control group to evaluate the anti-inflammatory effects of ORZ-NPs.

### 2.12. Statistical Analysis

All data are presented as mean ± SD from three independent experiments, each performed in triplicate. Statistical analyses were conducted using one-way analysis of variance (ANOVA) or two-way ANOVA, followed by Tukey’s post hoc test for multiple comparisons. Differences among treatment groups were assessed using Prism 9.4.1 software, and a *p*-value of less than 0.05 was considered statistically significant.

## 3. Results

### 3.1. Formulation and Physical Characterization of ORZ-NPs

**Figure 1:**
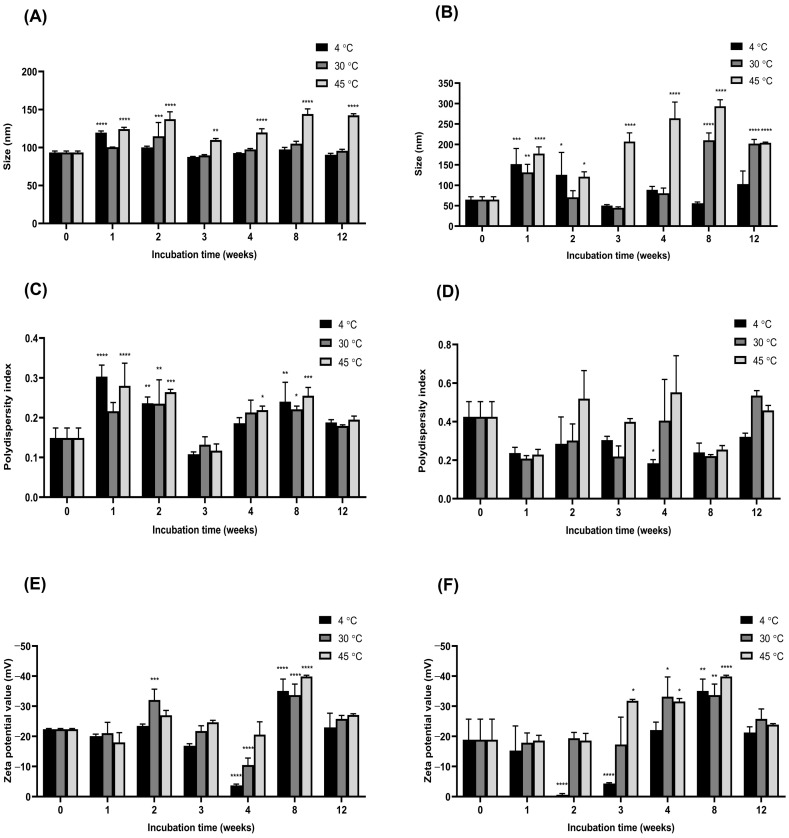
Physicochemical parameters of the ORZ-NPs and blank NPs. Particle size of the ORZ-NPs (**A**) and blank NPs (**B**), polydispersity index (PDI) values of the ORZ-NPs (**C**) and blank NPs (**D**), and zeta potential values of the ORZ-NPs (**E**) and blank NPs (**F**) were measured after fresh preparation and following storage for 1, 2, 3, 4, 8, and 12 weeks at 4 °C, 30 °C, and 45 °C (*n* = 3). Data demonstrate stability of particle size, PDI values, and zeta potential values over time under different storage conditions. Statistical significance compared to week 0: * *p* < 0.05, ** *p* < 0.01, *** *p* < 0.001, and **** *p* < 0.0001. Abbreviations: °C, degree Celsius; mV, millivolt; *n*, number; nm, nanometer; NPs, nanoparticles; ORZ-NPs, gamma oryzanol encapsulated nanoparticles; PDI, polydispersity index.

The ORZ-NPs were successfully developed using the nanoprecipitation method, employing 0.1% (*w*/*v*) Poloxamer 407 and 10% (*v*/*v*) PEG as stabilizers. The colloidal characteristics, including particle size, PDI, and zeta potential, are presented in Figure 1. The DLS size distribution profiles for all NP samples are exhibited in Supplementary Figure S1. Freshly prepared ORZ-NPs exhibited an average particle size of 93.320 ± 2.027 nm, a low PDI of 0.149 ± 0.025, and a zeta potential of −22.400 ± 0.252 mV, indicating a uniform and stable nanoparticle formulation. In comparison, the blank NPs had an average size of 64.740 ± 7.240 nm, a PDI of 0.425 ± 0.079, and a zeta potential of −18.900 ± 6.850 mV.

The physical characteristics of ORZ-NPs were assessed under different storage conditions (4 °C, 30 °C, and 45 °C) over 12 weeks (Figure 1A,C,E). At 4 °C, the particle size ranged from 87.640 ± 0.540 nm to 119.6 ± 2.17 nm, with a significant increase observed during the first week compared to the freshly prepared sample, but no further changes thereafter. The PDI values remained within 0.108 ± 0.006 to 0.300 ± 0.029, while the zeta potential ranged from −35.100 ± 3.930 to −3.730 ± 0.455 mV. At 30 °C, the particle size ranged from 89.580 ± 0.996 nm to 114.8 ± 18.03 nm, the PDI values varied from 0.132 ± 0.020 to 0.235 ± 0.060, and the zeta potential ranged from −33.700 ± 3.700 to −10.500 ± 2.330 mV. In contrast, storage at 45 °C resulted in a significant increase in particle size, with PDI values ranging from 0.117 ± 0.017 to 0.280 ± 0.057, and the zeta potential values varying from −39.900 ± 0.404 to −18.000 ± 3.260 mV. The ORZ-NPs exhibited good stability at 4 °C, with minimal changes in particle size and size distribution during the storage period.

The physical characteristics of the blank NPs were also evaluated under different storage conditions (4 °C, 30 °C, and 45 °C) over 12 weeks (Figure 1B,D,F). At 4 °C, the particle size ranged from 50.180 ± 2.632 nm to 151.700 ± 38.32 nm, with a significant increase observed during the first two weeks compared to the freshly prepared sample, but no notable changes were observed thereafter. The PDI values varied from 0.184 ± 0.019 to 0.425 ± 0.079, with a significant decrease noted at week 4. The zeta potential ranged from −35.100 ± 3.930 mV to −0.633 ± 0.424 mV, showing significant reductions at week 2 and week 3 and a significant increase at week 8. At 30 °C, the particle size ranged from 50.180 ± 2.632 nm to 151.700 ± 38.320 nm, with a significant increase observed at week 1, week 8, and week 12. The PDI values varied from 0.208 ± 0.016 to 0.535 ± 0.026, with a significant reduction at week 4. The zeta potential ranged from −33.700 ± 3.700 mV to −17.300 ± 9.100 mV, with significant increases at week 4 and week 8. At 45 °C, the particle size increased significantly compared to the freshly prepared sample, ranging from 44.790 ± 2.459 nm to 201.800 ± 10.560 nm. The PDI values ranged from 0.255 ± 0.021 to 0.552 ± 0.190. The zeta potential ranged from −39.900 ± 0.404 mV to −18.600 ± 2.400 mV, with significant elevations observed at week 3, week 4, and week 8. These results indicate that the blank NPs are most stable at 4 °C, with increased particle size, PDI, and zeta potential variability observed at higher temperatures, particularly at 45 °C.

**Figure 2:**
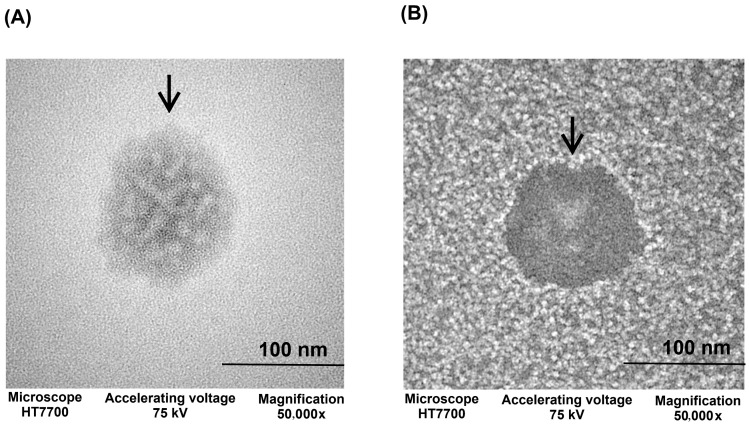
TEM image of the ORZ-NPs (**A**) and blank NPs (**B**), showing nanoparticle morphology and size. The black arrows indicate the observed nanoparticles. Abbreviations: kV, kilovolt; nm, nanometer; NPs, nanoparticles; TEM transmission electron microscopy.

Morphology of the ORZ-NPs and blank NPs was observed through TEM (Figure 2). The ORZ-NPs displayed a spherical shape and a uniform appearance. Similarly, the blank NPs exhibited a morphology comparable to that of the ORZ-NPs.

### 3.2. Chemical Stability and Chromatographic Analysis of ORZ-NPs

The stability of ORZ in NPs was assessed over 90 days under 4 °C, 30 °C, and 45 °C. Mean ORZ concentration and % remaining are exhibited in Table 1. Over the 90 days, the ORZ concentration remained relatively stable, with minor fluctuations observed under three storage conditions. At 4 °C, ORZ concentration increased slightly, reaching 1.759 ± 0.031 mg mL^−1^ by day 90, corresponding to 101.791% of the initial concentration, indicating enhanced stability at lower temperatures. At 30 °C, the mean concentration of ORZ also increased slightly, reaching 1.754 ± 0.041 mg mL^−1^ by day 90, equivalent to 101.478% of the initial concentration, reflecting consistent reproducibility of the measurements. At 45 °C, a slight decrease in ORZ concentration was observed, with the levels declining to 1.074 ± 0.006 mg mL^−1^ by day 90, equivalent to 98.610% of the initial concentration, reflecting consistent analytical reproducibility at higher temperatures. These findings confirmed that ORZ concentration in NPs maintains excellent stability across varying temperatures (4 °C, 30 °C, and 45 °C) over 90 days, highlighting its potential suitability for extended storage in practical applications.

**Figure 3:**
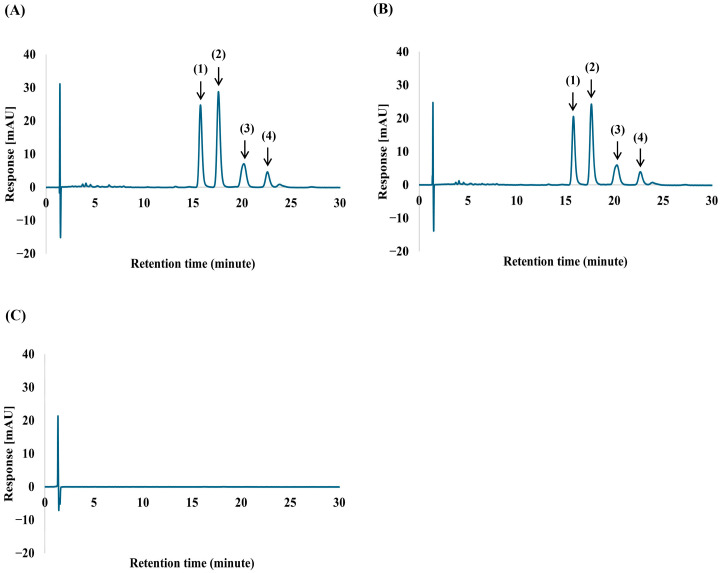
HPLC chromatogram of ORZ standard (**A**), ORZ-NPs (**B**), and blank NPs (**C**), showing the major ORZ compounds. Peaks are identified as follows: (1), cycloartenyl ferulate; (2), methylenecycloartanyl ferulate; (3), campesteryl ferulate; (4), β-sitosteryl ferulate. Abbreviations: HPLC, high performance liquid chromatography; mAU, milli-absorbance unit; NPs, nanoparticles; ORZ-NPs, gamma oryzanol encapsulated nanoparticles.

The chromatographic analysis of ORZ and its four isomers, including cycloartenyl ferulate, methylenecycloartanyl ferulate, campesteryl ferulate, and β-sitosteryl ferulate remained consistent throughout the study period. The retention times for these components were stable across all time points, as illustrated in Figure 3A,B. The chromatogram of the blank NPs (Figure 3C) validated the method’s specificity, showing no interfering peaks at the retention times corresponding to the ORZ components.

### 3.3. In Vitro Release Kinetics of ORZ-NPs

**Figure 4:**
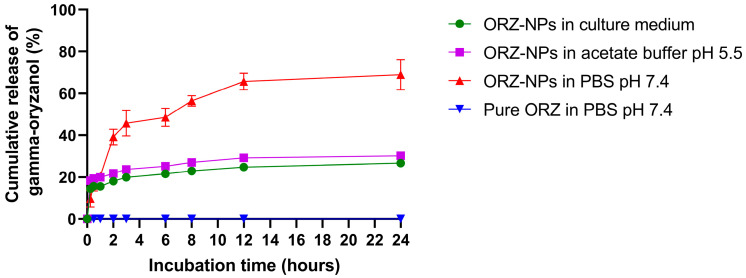
Cumulative release (%) of ORZ from ORZ-NPs and ORZ powder over 24 h (*n* = 3), showing the enhanced release profile of the nanoparticle formulation. Abbreviations: *n*, number; ORZ, gamma-oryzanol; ORZ-NPs, gamma oryzanol encapsulated nanoparticles; PBS, phosphate buffered saline.

Figure 4 illustrates the cumulative release profile of ORZ (%) from NPs over 24 h. In PBS pH 7.4, the ORZ-NPs formulation exhibited a biphasic release pattern. An initial burst release was observed during the first 6 h, with approximately 50% of ORZ released, suggesting the presence of loosely bound or surface-associated drug molecules. Subsequently, the release rate decreased, transitioning into a sustained release phase that continued until 12 h, after which the curve plateaued at approximately 70% cumulative release. The plateau indicated that most of the ORZ had been released, with any remaining fraction likely trapped within the NPs matrix. This release profile demonstrated the potential of the NPs system to deliver an initial effective dose while maintaining prolonged ORZ availability, an advantageous feature for controlled drug delivery applications. The maintenance of sink conditions through medium replacement ensured reliable measurement of release kinetics throughout the study. In contrast, ORZ powder in PBS (pH 7.4) exhibited lower drug release compared to ORZ-loaded NPs. Furthermore, ORZ-NPs exhibited sustained release profiles in both culture medium without additional supplements and acetate buffer, with cumulative release reaching approximately 25–30% over 24 h (Figure 4). Kinetic analysis demonstrated that the release data fit the Higuchi model (R^2^ = 0.8886) (Supplementary Figure S2) and Korsmeyer–Peppas model (R^2^ = 0.9345), with a release exponent (n ≈ 0.44) (Supplementary Figure S3).

### 3.4. Cell Viability of RAW 264.7 Macrophages Exposed to ORZ-NPs

**Figure 5:**
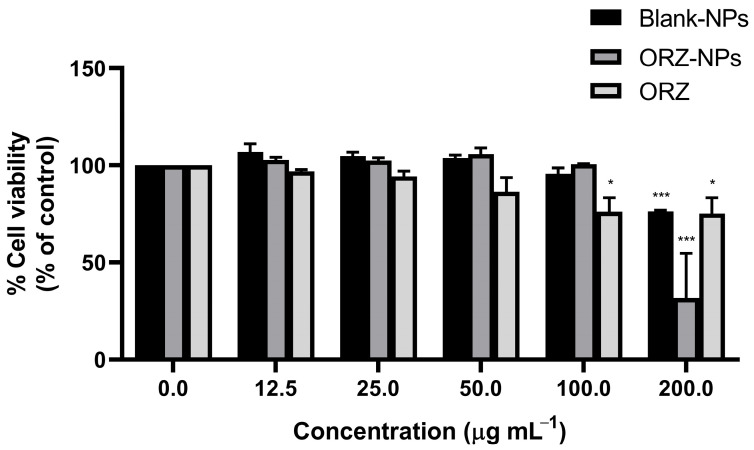
Cell viability of RAW 264.7 macrophages treated with ORZ-NPs, blank NPs, or ORZ powder at 0–200 μg mL^−1^ for 48 h, assessed by MTT assay. Cell viability is presented as the percentage of viable cells relative to the untreated, LPS-stimulated control (set as 100% viability). Data are expressed as the mean ± SD of three independent experiments (*n* = 3). Statistical significance: * *p* < 0.05 and *** *p* < 0.001. Abbreviations: LPS, lipopolysaccharide; mL, milliliter; MTT, 3-(4,5-dimethylthiazol-2-yl)-2,5-diphenyltetrazolium bromide; *n*, number; NPs, nanoparticles; ORZ, gamma-oryzanol; ORZ-NPs, gamma oryzanol encapsulated nanoparticles; SD, standard deviation; μg; microgram.

The cytotoxic effects of ORZ-NPs, blank NPs, or ORZ powder on RAW 264.7 macrophages were evaluated using the MTT assay. The cell viability data, expressed as a percentage relative to the untreated, LPS-stimulated control, are displayed in Figure 5. Cell viability of ORZ-NPs, blank NPs, and ORZ remained above 80% at concentrations between 12.5 and 50 μg mL^−1^, suggesting minimal cytotoxicity at these concentrations. At 100 μg mL^−1^, a slight reduction in cell viability was observed for both ORZ-NPs and blank NPs. A more notable decrease was observed at the highest concentration (200 μg mL^−1^), the blank NPs maintained cell viability above 70%, while the ORZ-NPs reduced cell viability to below 50%. Whereas cell viability after incubation with ORZ powder at a concentration of 100 and 200 μg mL^−1^ was significantly reduced. Overall, the MTT assay results indicated that the ORZ-NPs are relatively non-toxic to RAW 264.7 macrophages at concentrations up to 50 μg mL^−1^, with cytotoxic effects observed at higher concentrations (100 and 200 μg mL^−1^). The 20% inhibitory concentration, selected for subsequent experiments, was set below 100 μg mL^−1^.

### 3.5. Effect of ORZ-NPs on iNOS Protein Expression Levels in LPS-Stimulated RAW 264.7 Macrophages

**Figure 6:**
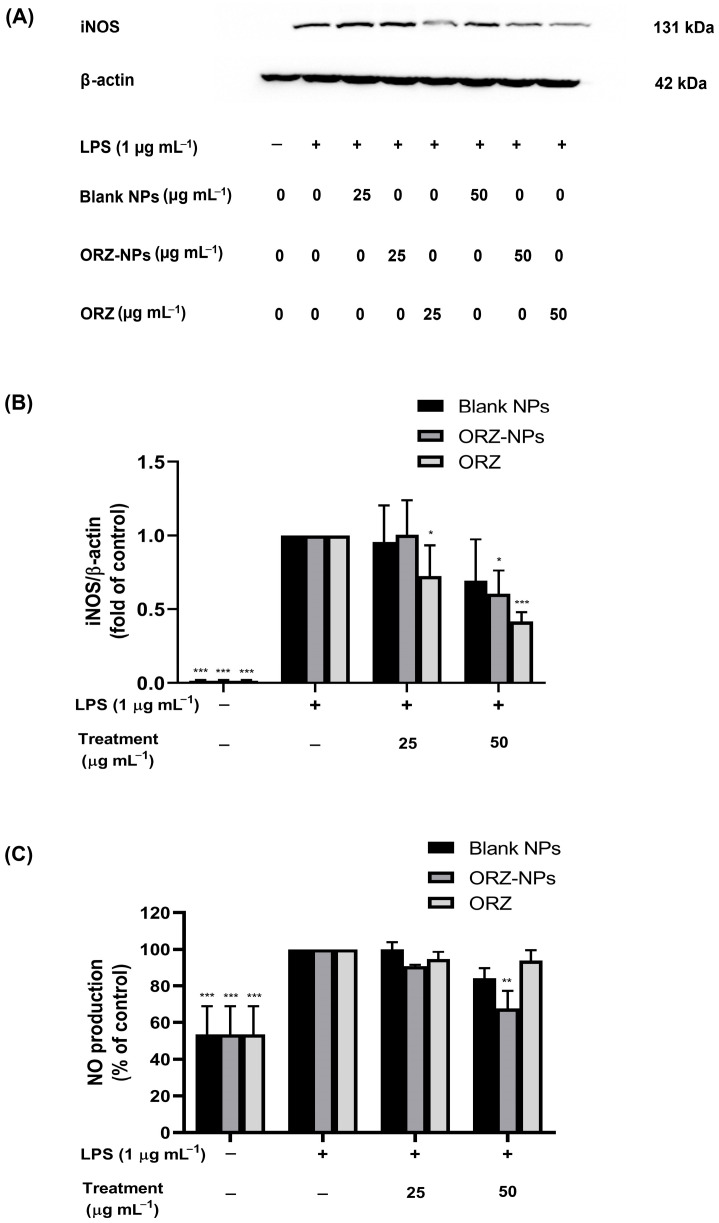
Inhibitory effects of the ORZ-NPs on iNOS protein expression levels and NO production in LPS-stimulated RAW 264.7 macrophages. Cells were pre-treated with blank NPs, ORZ-NPs, or ORZ (0–50 µg mL^−1^) for 1 h, followed by LPS stimulation (1 µg mL^−1^) for 24 h. Representative Western blot showing iNOS protein expression levels, with β-actin as a loading control (**A**). Quantification of iNOS protein expressed as fold change relative to the untreated, LPS-treated control. iNOS was normalized to β-actin and derived from three independent experiments (**B**). NO production measured by Griess assay, presented as percentage of the untreated, LPS-stimulated control (**C**). Data are expressed as mean ± SD (*n* = 3). Statistical significance: * *p* < 0.05, ** *p* < 0.01, and *** *p* < 0.001. Abbreviations: h, hour; iNOS, inducible nitric oxide synthase; kDa, kilodalton; LPS, lipopolysaccharide; mL, milliliter; *n*, number; NO, nitric oxide; NPs, nanoparticles; ORZ, gamma-oryzanol; ORZ-NPs, gamma oryzanol encapsulated nanoparticles; SD, standard deviation; μg, microgram.

iNOS protein expression levels were quantified and are presented in Figure 6A,B, demonstrating that induction of LPS (1 μg mL^−1^) significantly elevated iNOS protein expression levels compared to the non-LPS-stimulated group. In Figure 6A, the LPS-stimulated condition without treatment and LPS-stimulated conditions incubated with blank-NPs (25 and 50 µg mL^−1^) showed similar band intensities in the raw blot images. This suggests that blank NPs may contribute to background cellular responses comparable to LPS stimulation alone. When comparing blank NPs with ORZ powder or ORZ-NPs, the observed differences in band intensity arise from the differences in compositions of the formulations. The ORZ powder and ORZ-NPs contain the bioactive compound, which may modulate iNOS expression, whereas the blank NPs contain only the carrier material without ORZ. ORZ and ORZ-NPs may exhibit their effects through distinct mechanisms related to cellular uptake and bioavailability. Importantly, quantitative analysis (Figure 6B) normalizes iNOS protein expression levels relative to β-actin across three independent experiments and provides a more reliable assessment than visual inspection of a single representative blot.

### 3.6. Inhibition of LPS-Induced NO Production by ORZ-NPs

As shown in Figure 6C, LPS treatment alone significantly increased NO production, which was set as the control (100%). Pre-treatment with the ORZ-NPs at 25 µg mL^−1^ showed a trend toward decreased NO levels, although the reduction was not statistically significant. At 50 µg mL^−1^, the ORZ-NPs significantly reduced NO production to approximately 65% of the untreated, LPS-treated control (*p* < 0.01). In contrast, the blank NPs and ORZ did not significantly affect NO production at either concentration, indicating that the observed reduction in NO production was attributable to the active compound, ORZ, released from NPs rather than the NPs carrier. These findings demonstrated that the ORZ-NPs effectively reduced LPS-induced NO production in RAW 264.7 macrophages, suggesting potential anti-inflammatory properties.

### 3.7. Modulation of TNF-α and IL-6 Production by ORZ-NPs in LPS-Stimulated RAW 264.7 Macrophages

**Figure 7:**
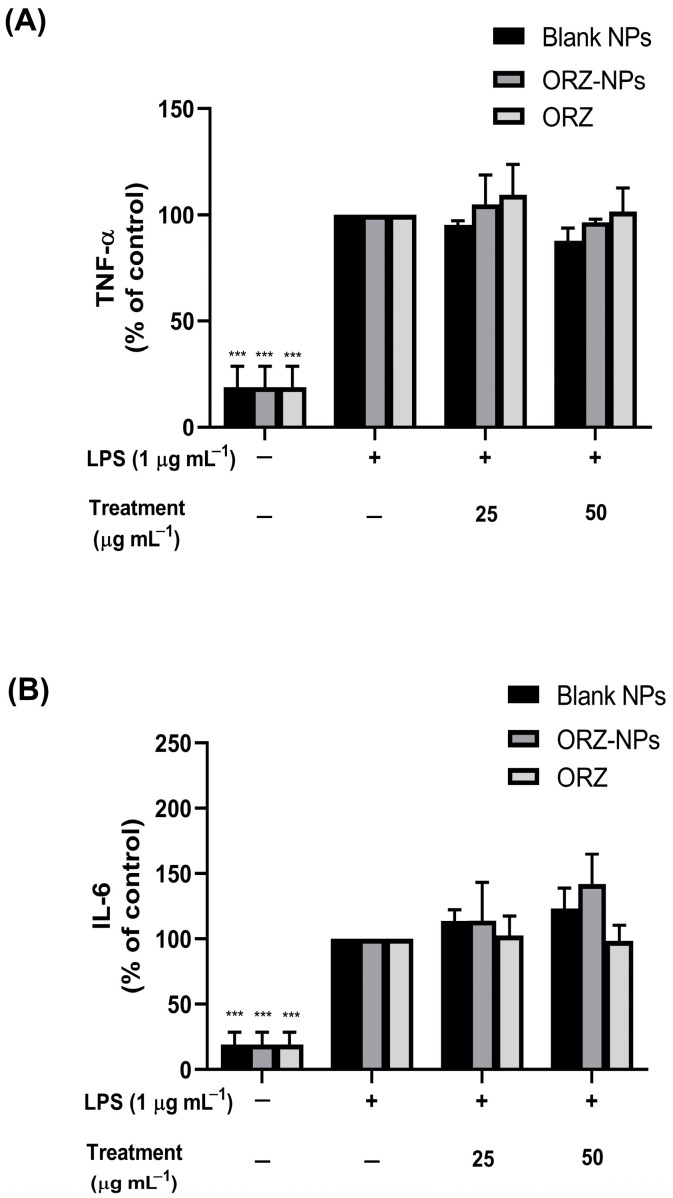
Effect of the ORZ-NPs on the secretion of pro-inflammatory cytokines, including TNF-α (**A**) and IL-6 (**B**) in LPS-stimulated RAW 264.7 macrophages. Cells were pre-treated with blank-NPs, ORZ-NPs, or ORZ powder (0–50 µg mL^−1^) for 1 h, followed by LPS stimulation (1 µg mL^−1^) for 24 h. Cytokine levels were measured by ELISA assay and are presented as percentages relative to the untreated, LPS-stimulated control. Data are shown as mean ± SD of three independent experiments (*n* = 3). Statistical significance: *** *p* < 0.001. Abbreviations: ELISA, enzyme-linked immunosorbent assay; h, hour; IL-6, interleukin-6; LPS, lipopolysaccharide; mL, milliliter; *n*, number; NPs, nanoparticles; ORZ, gamma-oryzanol; ORZ-NPs, gamma oryzanol encapsulated nanoparticles; SD, standard deviation; TNF-α, tumor necrosis factor-alpha; μg, microgram.

Figure 7A demonstrates that LPS significantly enhanced TNF-α secretion. Treatment with 25 µg mL^−1^ of the ORZ-NPs and ORZ maintained TNF-α levels comparable to the untreated, LPS-stimulated control, while treatment with 50 µg mL^−1^ of both ORZ-NPs and ORZ similarly did not affect TNF-α levels. As shown in Figure 7B, LPS stimulation significantly elevated IL-6 secretion. Pre-treatment with 25 µg mL^−1^ ORZ-NPs and ORZ led to a non-significant increase in IL-6 levels compared to the untreated, LPS-induced control group. Additionally, a higher concentration of 50 µg mL^−1^ ORZ-NPs and ORZ maintained IL-6 levels close to those of the control. Overall, these data suggest that TNF-α and IL-6 levels were unaffected by the various concentrations of ORZ-NPs.

## 4. Discussion

This study successfully formulated and characterized ORZ-NPs while investigating their anti-inflammatory potential in LPS-stimulated RAW 264.7 macrophages. The ORZ-NPs significantly enhanced the stability and bioavailability of ORZ while providing a controlled release mechanism, resulting in sustained therapeutic effects. Pre-treatment with the ORZ-NPs significantly reduced iNOS protein and NO production. However, TNF-α and IL-6 levels remained unchanged. These findings suggest a partial modulatory effect on inflammatory signaling pathway.

The ORZ-NPs, formulated using phospholipid-based phosphatidylcholine, yielded a particle size of approximately 93.32 ± 2.03 nm, which is consistent with prior studies that reported ORZ nanoparticle sizes ranging from 101 to 120 nm [26,27]. The variation in particle size compared to other NPs formulations, such as nanocurcumin (approximately 136 nm), may be attributed to variations in active compounds [22]. Smaller particle sizes are advantageous for enhancing cellular uptake and improving delivery efficiency [28]. In our study, the formulated ORZ-NPs demonstrated a PDI value of 0.149 ± 0.025, indicating good uniformity, as a PDI value below 0.1 is typically considered monodisperse [29], whereas values exceeding 0.7 suggest a very broad particle size distribution [30]. Similarly, a related study reported a PDI of 0.150 for ORZ liposomes [26], while another study observed a much lower PDI of 0.08 [27]. To improve the stability of NPs, Poloxamer 407 was incorporated as a nonionic surfactant to prevent aggregation, and PEG was added to increase hydrophilicity [31,32]. While PEG enhances colloidal stability, it may reduce cellular uptake and affect drug release efficiency [28]. The zeta potential of NPs is a critical indicator of their stability and surface charge. Our findings revealed that the zeta potential was around −22.400 ± 0.252 mV, consistent with previous reports of zeta potential values between −22 and −59 mV for ORZ-NPs [27]. Although nanoparticles with zeta potentials exceeding ±30 mV are considered highly stable due to strong repulsive forces, the values near neutrality (−10 to +10 mV) indicate limited stability [33]. Collectively, these data demonstrate that the formulation achieved a well-defined NPs profile suitable for reliable bioactivity.

The long-term stability of NPs is crucial for maintaining their biological efficacy, particularly under varying storage conditions [23,34]. In this study, we monitored the physicochemical properties of ORZ-NPs over 12 weeks at 4 °C, 30 °C, and 45 °C. While the formulation remained relatively stable at 4 °C, higher temperatures led to moderate changes in particle size and zeta potential. Consistent with a prior study, particle sizes of ORZ encapsulation remained stable at 4 °C and 25 °C, over 30 days [35]. In this study, the temporal increase and subsequent decrease in particle size observed during storage likely reflect a dynamic equilibration process typical of lipid-based colloidal systems. Initial size enlargement may arise from progressive hydration of the lipid matrix and transient swelling following water penetration into interfacial lipid regions, particularly under elevated temperature conditions that enhance lipid bilayer fluidity [36]. This hydration-driven expansion may also promote temporary soft aggregation between particles, contributing to short-term size increases. Over time, the lipid components undergo structural reorganization toward a thermodynamically more stable configuration, resulting in tighter lipid packing and partial contraction of particle size [37]. A gradual increase in particle size at 45 °C was observed for both formulations, likely due to thermal stress-induced particle association [38]; however, ORZ-NPs exhibited greater size stability than blank NPs, suggesting a stabilizing effect of ORZ incorporation. Transient changes in PDI at early time points were consistent with post-storage equilibration and remained within acceptable ranges for colloidal stability. Minor fluctuations in zeta potential were also observed, as expected for aqueous systems [38]. Although some parameters showed statistical differences compared with week 0, the absolute changes remained within ranges indicative of overall physicochemical stability, with no evidence of aggregation or formulation failure.

HPLC analysis confirmed that the ORZ-NPs exhibited enhanced stability of ORZ under various storage conditions. The retention of ORZ in the NPs protected it from environmental degradation, consistent with previous findings [15]. The release kinetics of ORZ-NPs in PBS (pH 7.4) revealed a biphasic release profile, with approximately 50% of ORZ released within 6 h, transitioning to a plateau of ~70% at 12 h. However, discrepancies with electrospray ORZ-loaded gliadin particles, which exhibited a faster initial release (approximately 60% at 180 min) [39], could be attributed to differences in the NP composition and formulation [40]. The controlled release highlights the potential of these NPs for sustained therapeutic applications. Compared with the release observed in PBS (pH 7.4), the overall release rate of ORZ was slower in both culture medium without additional supplements and acetate buffer pH 5.5, indicating that drug release is governed not only by external pH but also by NP structural dynamics and medium composition. Given the highly lipophilic nature of ORZ and its negligible ionization across physiological and mildly acidic pH ranges, the reduced release at pH 5.5 is unlikely to result from changes in ORZ solubility but rather from pH-dependent alterations in lipid packing behavior. Under mildly acidic conditions, partial protonation of ionizable lipid components may enhance lipid–lipid interactions [41], leading to a more ordered and compact internal lipid matrix that restricts diffusion of the hydrophobic drug molecules. In contrast, at physiological pH (7.4), predominantly neutral lipid species maintain relatively higher membrane fluidity [42], facilitating diffusion of ORZ from the nanoparticle core into the surrounding medium. Importantly, both acetate buffer (pH 5.5) and culture medium partially mimic intracellular or inflammation-associated microenvironments, suggesting that ORZ-NPs maintain controlled and stable release behavior under biologically relevant conditions. These findings support the suitability of ORZ-NPs for sustained delivery in cellular and inflammatory settings [43].

According to the Korsmeyer–Peppas model, the calculated release exponent (n ≈ 0.44) indicates Fickian diffusion-controlled transport, where drug release is primarily governed by molecular diffusion through the lipid matrix rather than by matrix erosion or swelling mechanisms [44]. Although lipid-based nanoparticles may involve combined processes such as structural relaxation or hydration effects, the obtained n value confirms that diffusion represents the dominant release mechanism in the present formulation. These findings collectively demonstrate that ORZ release from the NPs is mainly regulated by diffusion through the lipid matrix, supporting their suitability for sustained drug delivery applications.

The relatively higher variability observed in the MTT assay, particularly at higher concentrations, likely reflects the inherent biological heterogeneity of RAW 264.7 macrophages under inflammatory stimulation, biomolecule exposure, and nanoparticle exposure. At elevated concentrations, differences in cellular uptake and mitochondrial activity can contribute to increased variability in metabolic readouts. Furthermore, previous reports have demonstrated that biomolecules such as ascorbic acid, tocopherols, glutathione, glutathione S-transferase, and cysteine can directly reduce MTT [45]. Similarly, exposure to NP at high concentrations can also induce MTT reduction [46]. Taken together, these factors can influence MTT readouts. All experiments were performed in three independent biological replicates (*n* = 3), and despite this variability, the overall trends were consistent and statistically significant, supporting the robustness of the results.

The anti-inflammatory effects of the ORZ-NPs were demonstrated by their ability to inhibit LPS-induced iNOS protein expression and reduce NO production. These findings align with previous studies showing that ORZ downregulated iNOS expression and suppressed NO synthesis, thereby alleviating chronic inflammation and protecting cardiovascular health [10,11,12]. Assessing NO production can serve as a useful marker for evaluating anti-inflammatory effects in LPS-stimulated RAW 264.7 macrophages, as supported by evidence from systematic reviews and meta-analyses [47]. NO plays a dual role in inflammation and cardiovascular health. It is produced by endothelial cells, promoting vasodilation and preventing platelet aggregation, essential for vascular homeostasis. However, excessive NO production during inflammation can contribute to tissue damage and endothelial dysfunction, impairing vascular tone regulation and increasing the risk of atherosclerosis and cardiovascular complications [48,49]. Modulating iNOS expression is a key strategy to limit NO accumulation and mitigate these adverse effects, as iNOS overexpression is implicated in inflammatory diseases such as atherosclerosis and myocardial infarction [50,51]. Notably, previous studies have demonstrated that both cycloartenyl ferulate, a major bioactive component of ORZ, and ORZ itself act as key modulators of inflammation by inhibiting iNOS expression through the suppression of NF-κB [52,53]. However, NF-κB activation was not evaluated in the present study; therefore, further investigations are required to clarify the underlying molecular mechanisms and strengthen the interpretation of these findings. By inhibiting iNOS and modulating NO levels, the ORZ-NPs may provide targeted therapeutic benefits in inflammation-related conditions.

In this study, although ORZ-NPs significantly modulated iNOS and NO production, ORZ-NPs did not significantly reduce TNF-α and IL-6 production (Figure 7). These findings contrast with prior studies in which rice bran oil, rich in ORZ and tocopherol, effectively reduced these cytokines in macrophages [10,11,54]. This discrepancy suggests that the anti-inflammatory effects of ORZ-NPs may be pathway-specific rather than broad-spectrum. These findings may be explained by the temporal regulation of inflammatory signaling following LPS stimulation. Activation of Toll-like receptor 4 primarily triggers the MyD88-dependent pathway, leading to rapid induction of pro-inflammatory cytokines via MAPK and NF-κB signaling [55]. TNF-α secretion typically begins within a few hours after LPS stimulation, starting at approximately 4 h and reaching peak levels at approximately 16 h [56]. Whereas IL-6 production generally peaks within approximately 8–12 h post-stimulation with LPS [57]. Given that our experimental design involved a 1 h pre-treatment followed by co-incubation, cytokine release may have occurred before ORZ-NPs achieved sufficient cellular uptake and intracellular drug release to effectively suppress early cytokine production. Once secreted, these cytokines remain stable in the culture supernatant, which may obscure potential late inhibitory effects when measured at the 24 h time point. In contrast, inflammatory mediators such as iNOS-derived NO are regulated through later-phase signaling requiring de novo protein synthesis, with maximal accumulation typically observed several hours after stimulation [58]. This extended timeframe likely allows greater nanoparticle internalization and pharmacological activity, which may explain the more pronounced inhibitory effect of ORZ-NPs on the iNOS/NO pathway compared with early-response cytokines such as TNF-α and IL-6.

This study has some limitations that should be acknowledged. First, only two concentrations of ORZ-NPs (25 and 50 µg mL^−1^) were evaluated, based on preliminary cytotoxicity data. While these concentrations fall within a non-toxic and potentially bioactive range for RAW 264.7 macrophage cells, a full dose-response analysis was not conducted. This limits our ability to determine the optimal therapeutic window and the highest safe dose. Future studies should examine a broader range of concentrations, particularly between 50 and 100 µg mL^−1^, to better define the threshold at which cytotoxicity begins while maintaining cell viability above 80%. Second, the current study did not include time-course analyses to capture the kinetics of anti-inflammatory effects. Although the release profile of ORZ-NPs indicates a sustained release over 12 h, we did not vary the treatment duration to assess corresponding changes in inflammatory mediators. Future investigations should incorporate multiple time points to better understand the temporal dynamics of ORZ-NP activity. Addressing these limitations will provide a more comprehensive understanding of the dose- and time-dependent effects of ORZ-NPs in inflammation. Third, although ORZ-NPs significantly reduced iNOS and NO production, these effects are presumed to be associated with cellular uptake by macrophages. However, direct visualization of NP internalization was not performed. Future studies should include imaging-based approaches to confirm intracellular localization and strengthen the mechanistic interpretation. Although in vivo studies would further enhance the translational relevance of these findings, the present study was designed as an in vitro formulation and mechanistic investigation to establish proof of concept prior to animal experimentation. Future studies will focus on validating the anti-inflammatory effects of ORZ-NPs in appropriate in vivo inflammation models. Finally, the influence of PEG 400 density on macrophage internalization was not investigated. Although PEG 400 was incorporated primarily as a stabilizing co-solvent rather than a surface-grafted stealth layer, its presence may still affect NP and cell interactions [59]. Optimization of PEG density to balance colloidal stability with efficient macrophage uptake warrants further investigation.

## 5. Conclusions

Our findings demonstrated that the ORZ-NPs treatment effectively reduces iNOS protein expression and NO production under inflammatory conditions, highlighting their potential as a partial therapeutic strategy for chronic inflammation and related conditions. Although the ORZ-NPs did not significantly reduce TNF-α and IL-6 production, their enhanced stability and bioavailability suggest promise as a nutraceutical candidate. Future research, including in vivo studies and clinical studies, is warranted to optimize treatment conditions and evaluate the broader therapeutic potential of the ORZ-NPs in managing chronic inflammation and inflammation-related conditions.

## Figures and Tables

**Table 1:** ORZ contents and percentage remaining in ORZ-NPs over 90 days under three storage conditions (4 °C, 30 °C, and 45 °C) (*n* = 3). Measurements were taken at day 0, 30, 60, and 90 to evaluate the stability of the nanoparticles.

Condition	Day	Mean Concentration ± SD (mg mL^−1^)	% Remaining ± SD	% RSD
Freshly prepare	0	1.728 ± 0.004	100.00	0.258
4 °C	30	1.764 ± 0.029	102.088 ± 1.686	1.651
	60	1.757 ± 0.033	101.695 ± 1.929	1.897
	90	1.759 ± 0.031	101.791 ± 1.809	1.777
30 °C	30	1.752 ± 0.035	101.395 ± 2.021	1.994
	60	1.752 ± 0.038	101.364 ± 2.171	2.141
	90	1.754 ± 0.041	101.478 ± 2.349	2.315
45 °C	30	1.707 ± 0.015	98.753 ± 0.856	0.867
	60	1.706 ± 0.024	98.698 ± 1.413	1.432
	90	1.704 ± 0.006	98.610 ± 0.343	0.348

Abbreviations: °C, degree Celsius; mg, milligram; mL, milliliter; *n*, number; ORZ, gamma-oryzanol; ORZ-NPs, gamma oryzanol encapsulated nanoparticles RSD, relative standard deviation; SD, standard deviation.

## Data Availability

Data sets used for this study are available from the corresponding author upon reasonable request.

## Supplementary Materials

The following supporting information can be downloaded at: https://www.mdpi.com/article/10.3390/pharmaceutics18030365/s1, Figure S1: Dynamic light scattering (DLS) size distribution by number for the (A) ORZ-NPs and (B) Blank NPs; Figure S2: Higuchi model fitting; Figure S3: Korsmeyer–Peppas model fitting; Figure S4: The original western blot images.

## Abbreviations

°C, degree Celsius;µg, microgram; μm, micrometer; µL, microliter; ANOVA, analysis of variance; CO_2_, carbon dioxide; DLS, dynamic light scattering; DMEM, Dulbecco’s modified eagle medium; DMSO, dimethyl sulfoxide; ECL, enhanced chemiluminescence; ELISA, enzyme-linked immunosorbent assay; HPLC, high-performance liquid chromatography; IgG, immunoglobulin G; IL-1, interleukin-1; IL-6, interleukin-6; iNOS, inducible nitric oxide synthase; kDa, kilodalton; kV, kilovolt; LPS, lipopolysaccharide; MAPK, mitogen-activated protein kinase; mg, milligram; mL, milliliter; mm, millimeter; MTT, 3-(4,5-dimethylthiazol-2-yl)-2,5-diphenyltetrazolium bromide; mV, millivolt; nm, nanometer; NF-κB, nuclear factor-kappa B; NO, nitric oxide; NPs, nanoparticles; ORZ, gamma-oryzanol; ORZ-NPs, gamma-oryzanol nanoparticles; PBS, phosphate buffered saline; PDI, polydispersity index; PEG, polyethylene glycol; SD, standard deviation; TBST, tris buffered saline with Tween; TEM, transmission electron microscopy; TNF-α, tumor necrosis factor-alpha; UV, ultra-violet; *v*, volume; *w*, weight.
