# Supplementary material for: Formulation of Gamma-Oryzanol Encapsulated Nanoparticles and Their Modulation Effects on Inducible Nitric Oxide Synthase and Nitric Oxide in LPS-Stimulated RAW 264.7 Macrophages
Source: Pharmaceutics. 2026 Mar 14;18(3):365. doi: 10.3390/pharmaceutics18030365 (PMC13029237; doi:10.3390/pharmaceutics18030365)
Supplement: Supplementary file 1 [file pharmaceutics-18-00365-s001.zip › pharmaceutics-4160686-supplementary.pdf]

Formulation of gamma-oryzanol encapsulated nanoparticles and their modulation effects on inducible nitric oxide synthase and nitric oxide in LPS-stimulated RAW 264.7 macrophages

Supporting Information

(A)

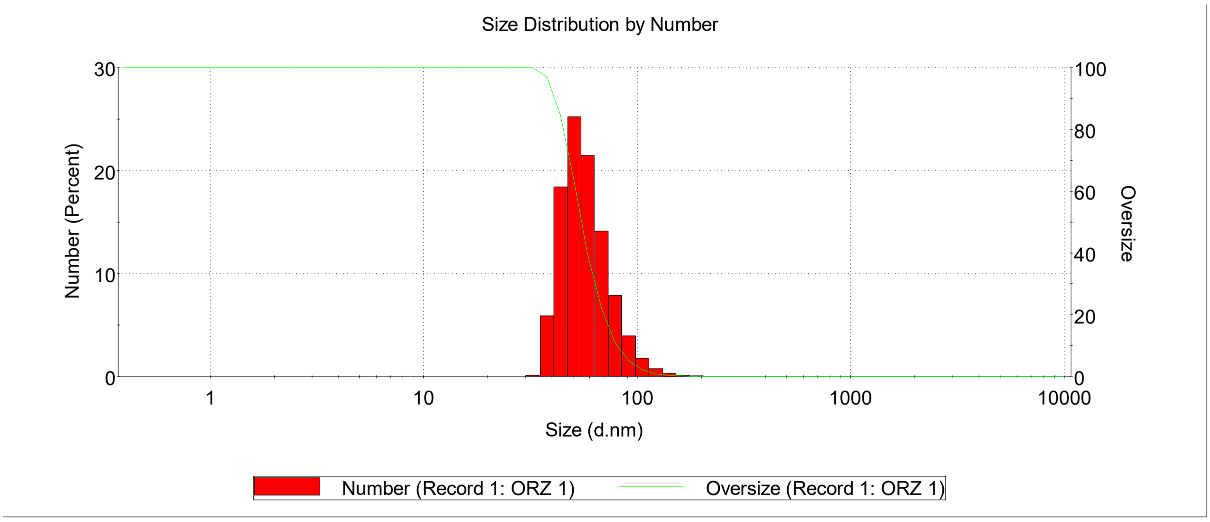

(B)

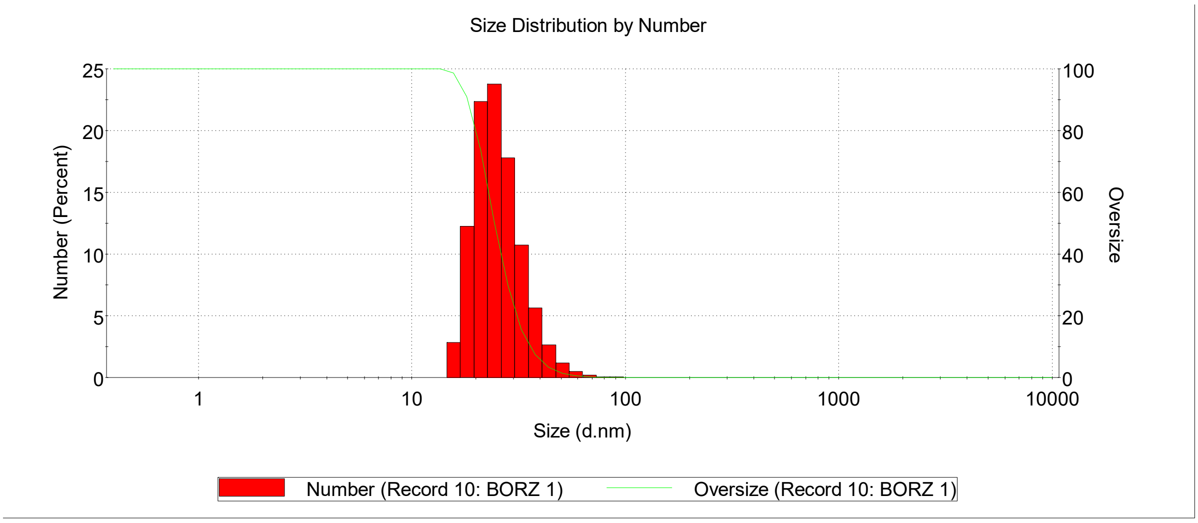

Supplementary Figure S1 Dynamic light scattering (DLS) size distribution by number for the (A) ORZ-NPs and (B) Blank NPs

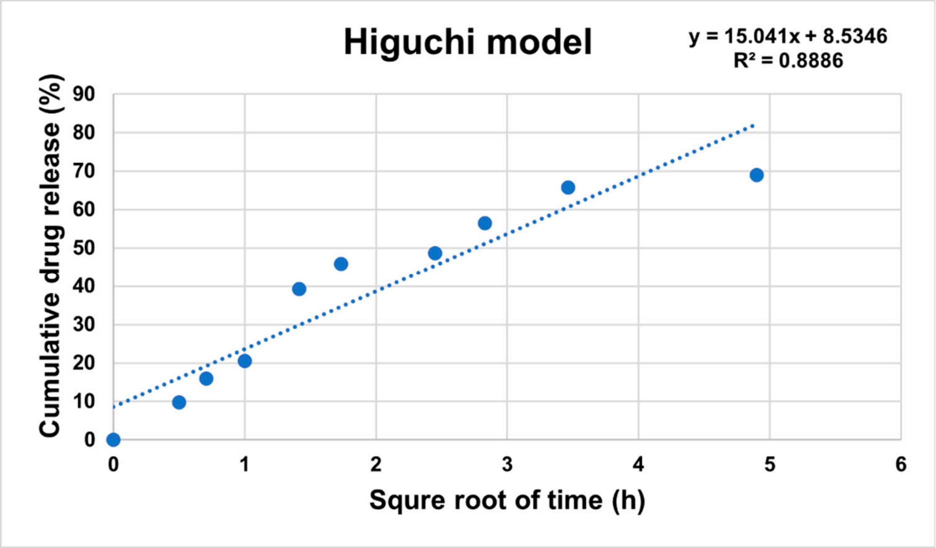

Supplementary Figure S2 Higuchi model fitting. Cumulative ORZ release from ORZ-loaded nanoparticles plotted as a function of the square root of time.

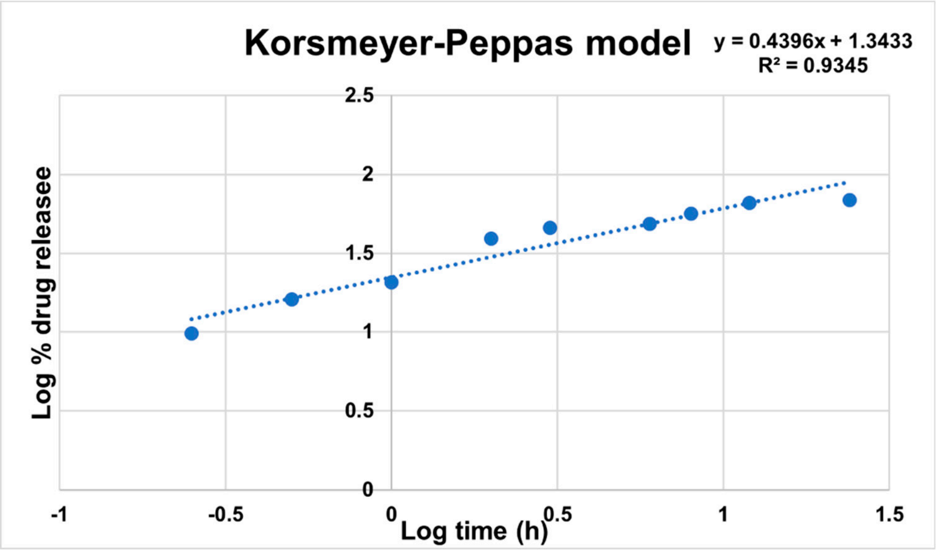

Supplementary Figure S3 Korsmeyer–Peppas model fitting. Korsmeyer-Peppas log-log plot of ORZ release.

Western blot results

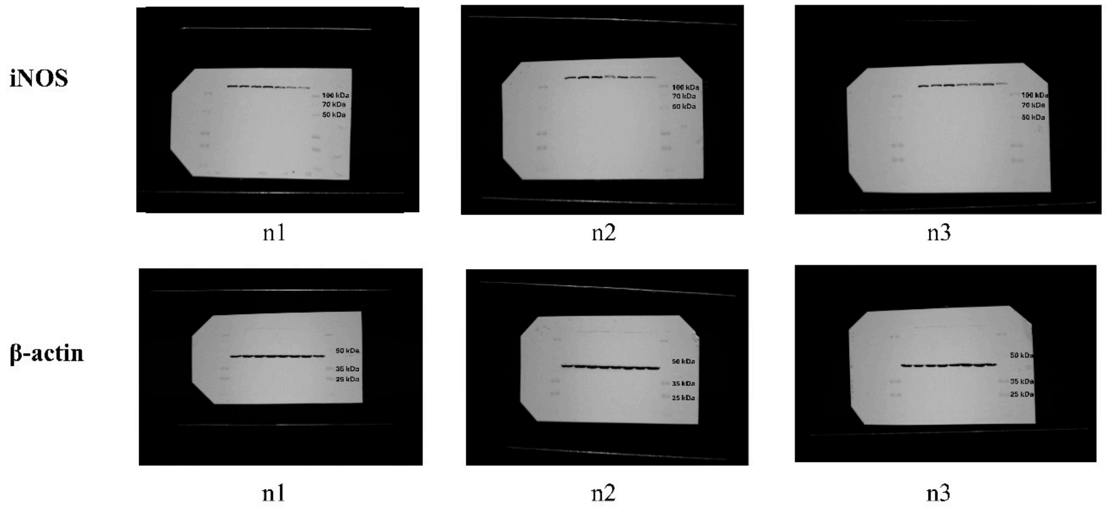

Supplementary Figure S4 The original western blot images.
